# RePAIR consensus guidelines: Responsibilities of Publishers, Agencies, Institutions, and Researchers in protecting the integrity of the research record

**DOI:** 10.1186/s41073-018-0055-1

**Published:** 2018-12-19

**Authors:** Collaborative Working Group from the conference “Keeping the Pool Clean: Prevention and Management of Misconduct Related Retractions”, Carolyn J. Broccardo, Noémie Aubert Bonn, Laura Bandura-Morgan, Jeffrey Beall, Catherine Bens, Monica Bradford, Lyndon Branfield, Arthur M. Buchberg, Ferric Fang, Anna Keith, Barb Houser, Jennifer K. Nyborg, Kelly Perry, Charon A. Pierson, Kenneth D. Pimple, Kaoru Sakabe, Dan Wainstock, Tamara Welschot, B. R. Woods, Alice Young

**Affiliations:** 1 Colorado State University, Fort Collins, USA; 2 Hasselt University, Hasselt, Belgium; 3 National Science Centre, Krakow, Poland; 4 Science/AAAS, Washington, DC, USA; 5 Springer Nature, London, United Kingdom; 6 American Association for Cancer Research, Philadelphia, PA USA; 7 University of Washington, Seattle, WA USA; 8 COPE: Committee on Publication Ethics, Eastleigh, United Kingdom; 9 Teach RCR Services, Bloomington, IN USA; 10 American Society for Biochemistry and Molecular Biology, Rockville, MD USA; 11 Harvard Medical School, Boston, MA USA; 12 Springer Nature, Dordrecht, Netherlands; 13 Texas Tech University, Lubbock, TX USA

**Keywords:** Research integrity, Retractions, Researchers, Publishers, Editors, Agencies, Institutions, Research misconduct, International, Communication

## Abstract

The progression of research and scholarly inquiry does not occur in isolation and is wholly dependent on accurate reporting of methods and results, and successful replication of prior work. Without mechanisms to correct the literature, much time and money is wasted on research based on a crumbling foundation. These guidelines serve to outline the respective responsibilities of researchers, institutions, agencies, and publishers or editors in maintaining the integrity of the research record. Delineating these complementary roles and proposing solutions for common barriers provide a foundation for best practices.

## Background

The iterative process of research allows science to continuously advance and progress, but this renders research vulnerable to inaccuracy and error. Given the frequent delays between identifying and correcting literature, inaccuracies may persist long after discovery [1].

Researchers, institutions, agencies, and publishers or editors have complementary roles and responsibilities in maintaining the integrity of the research record [2]. However, due to a myriad of real and perceived barriers to communication, the requisite interactions between these key stakeholders are often insufficient. This frequently leads to delays in correcting the literature and a lack of transparency regarding rationale for posted retractions. All of these problems diminish the public’s faith in the research process and must be communally addressed. The following guidelines define the respective responsibilities of key stakeholders when questions arise regarding possible research or publication misconduct and identify barriers to communication as well as potential solutions. This document serves to complement prior guidance from the Committee on Publication Ethics (COPE) and others for responding to published inaccuracies [3–6]. The existing literature provides advice on dealing with retractions, and on cooperation between different groups such as editors and universities. We aim to expand these recommendations by providing best practices for other key groups, and also address frequent barriers to effective communication and provide suggestions for working through these challenges.

The following guidelines emerged from the collaborative effort of a working group from the conference entitled Keeping the Pool Clean: Prevention and Management of Misconduct Related Retractions held on July 20–22, 2016, in Fort Collins, Colorado, USA. Collectively, this 20-member working group has expertise spanning multiple scientific and professional disciplines with representatives from 14 institutions and five countries, including attorneys, Research Integrity Officers, journal editors, publishers, federal officials, and researchers. These guidelines are intentionally US focused, but the general nature of these recommendations is equally applicable in an international context. The intent of these guidelines is not to provide prescriptive recommendations for every potential scenario, rather to outline best practices that can serve as a springboard for implementation strategies. As such, this is a purposefully concise outline of key stakeholder responsibilities.

## Main text

### Responsibilities

#### Researchers

1. Maintain compliance with, and foster an environment conducive to, the highest ethical standards for research including robust and rigorous research practices;
2. Address and communicate observations of likely ethical breaches as appropriate;
3. Sustain and create a local environment where ethical issues can be safely and honestly discussed;
4. Employ rigorous experimental and analytical methods;
5. Maintain careful and accurate research records of all primary data, protocols, and other procedures (including analyses, software/code version);
6. Archive data and documentation according to applicable guidelines, including those set by funding agencies and institutions;
7. Regularly review raw data with the group leader, those who created the data, and other interested parties;
8. Perform and report robust and transparent data analysis;
9. Provide primary data and documentation on request (in the case of research involving human participants, appropriate safeguards to ensure participant confidentiality must be in place);
10. Cooperate with government, institutional, and journal inquiries.

#### Institutions

1. Designate a Research Integrity Officer or equivalent administrative officer to ensure institutional research activities are in compliance with regulations and community standards;
2. Ensure prominent posting of contact information for designated Research Integrity Officer;
3. Create an environment that fosters ethical behavior and rigorous practice through effective mentoring, education, and institutional oversight;
4. Establish clear, confidential channels to report allegations of misconduct and protect whistleblowers from retaliation;
5. Perform thorough, timely, and impartial assessment and investigation of credible allegations of research misconduct in accordance with relevant rules, policies, and laws;
6. Protect both respondent and complainant privacy (to the extent possible) during ongoing investigations;
7. Provide findings (redacted according to institutional policy) upon request from investigations when misconduct is found;
8. Identify publications that warrant retraction or correction and provide timely notification and details to journals;
9. Cooperate in investigations and communicate with publishers and responsible government agencies as appropriate.

#### Publishers and editors

1. Effectively screen manuscripts for plagiarism, inappropriate textual and/or image duplications, discrepancies indicating inappropriate image manipulation, statistical irregularities, and other common pitfalls before publication;
2. Publish clear policy and process guidelines regarding research and publication misconduct;
3. Examine suspicions or allegations of serious problems in submitted manuscripts or published work (e.g., fraudulent data), beginning with a professional and open communication channel with the author(s), and, if appropriate, requesting primary data;
4. Notify institutions when misconduct involving a publication or submitted manuscript is suspected after consideration of the authors’ response; publishers should consider adding to their authorship policy a requirement for authors to supply appropriate contact information for their institutional representatives, such as the Research Integrity Officer, at the time of manuscript submission;
5. Determine which publications warrant retraction, expression of concern, or correction in accordance with Committee on Publication Ethics (COPE) guidelines [3];
6. Cooperate with institutional investigations involving allegations of research misconduct, including sharing of relevant information within publishers’ stated policy guidelines;
7. Issue freely available retraction/correction notices that provide a summary of the retraction, including who is retracting the article (e.g., author/s, institution, editor) and reason for retraction, in accordance with COPE guidelines [3];
8. Ensure retracted articles are clearly identified as such by search engines, and abstracting and indexing services.

#### (Regulatory or funding) agencies

1. Publically post contact information for reporting of misconduct concerns;
2. Where mandate allows, perform thorough, timely, and impartial oversight and/or investigation of credible allegations of misconduct according to relevant agency policy;
3. Assess appropriate penalties (including appropriate recommendations for correcting the research record) for those found guilty of research misconduct according to relevant mandates;
4. Ensure that relevant legal mandates and sanctions are executed per agency policy;
5. Notify public of findings of research misconduct according to applicable federal or agency policy.

## Overcoming barriers to communication: an agenda for harmonization

The responsibilities of researchers, institutions, agencies, and publishers in protecting the integrity of the research record are complementary and interdependent. In order to encourage collaboration, it is important to recognize the complexities and recurrent barriers to communication and to discuss potential solutions. The following challenges are frequently encountered in the communication process but should not be viewed as insurmountable barriers. We offer positive suggestions for moving through these roadblocks and outline opportunities for harmonization.

## Share information

Privacy policy and/or laws vary greatly among institution, agency, and country, which make it difficult to share protected information during and even after an investigation, regardless of the conclusion. Establishing and posting clear policies on how to handle allegations of research misconduct can serve to set reasonable expectations, especially regarding confidentiality. When possible, policies should be commonly agreed upon between institutions and journals to simplify legal permissions and to promote compatibility and legitimacy of policies. Examples of best practice recommendations for cooperation between journals and institutions are available and should be consulted [5, 6].

## Appropriately handle and prevent threats of legal action

The best defense against researchers or authors who threaten to sue is for institutions to carry out thorough and confidential investigations into allegations of research misconduct, while adhering to relevant federal and institutional policies. This includes establishing and carefully following procedures and policies for handling allegations of research misconduct.

## Establish adequate national oversight

Not all countries have a central agency or policy on how to handle allegations of research misconduct, although this is a topic of active conversation in Europe [7, 8]. Many U.S. agencies have avenues for reporting concerns of research misconduct (National Institutes of Health, National Science Foundation, Food and Drug Administration, Department of Agriculture), and other systems include Sweden’s independent Expert Group for Scientific Misconduct at the Central Ethical Review Board, and the Australian Research Integrity Committee, all of which could serve as a model for other groups to establish national mechanisms for reporting and investigating allegations of misconduct.

## Ensure institutional research integrity oversight

There is great variation in institutional regulations, reporting channels, and allegation processes. Institutions should designate a Research Integrity Officer or equivalent administrative officer to oversee compliance of research activities in accordance with institutional policy and community standards. Additionally, the Research Integrity Officer should establish clear, confidential channels to report allegations of research misconduct and protect whistleblowers from retaliation, and institutions should ensure prominent posting of the Research Integrity Officer’s contact information and whistleblowing procedures.

## Protect whistleblowers

Institutions and agencies must protect whistleblowers from retaliation [9].

## Maintain transparency and declare potential conflicts of interest

It is the duty of researchers, institutions, and journals to prevent conflicts of interest from interfering with the open and honest communication and/or investigation of allegations of research misconduct. Conflicts of interest could include financial interests and fear of reputational loss, among others. Minimizing and fully declaring potential conflicts of interests are essential to transparency.

## Create a positive research culture through researcher training in responsible conduct of research and good research practices

Institutions should facilitate a positive research culture [10, 11] at every level. Such a culture would be supported both by adopting hiring and promotion criteria that explicitly recognize and reward rigor, transparency, and ethical and collegial conduct, and by embedding responsible conduct of research (RCR) and good research practice within core training curriculum of every researcher, faculty member, and trainee. Such efforts may also include mandatory RCR training and/or the signing of an ethics code of conduct.

## Perform timely investigations

Investigating allegations of research misconduct constitutes a considerable time and cost commitment. By establishing a clear research misconduct policy and procedures and committing to timely communication between stakeholders, involved parties may be spared substantial time and resources.

## Conclusion

This document serves to summarize the respective roles of key stakeholders in maintaining the integrity of the research record and puts forth recommendations to overcome common communication barriers. It is important to note that many, if not most, errors in the published literature are not due to research misconduct, but rather to other factors including, but not limited to, irreproducible research, honest error, authorship disputes, or lack of necessary approvals (i.e., institutional human/animal/biosafety approval). We believe these recommendations will be useful not only to prevent research misconduct, but to promote open communication between stakeholders in sharing the responsibility to maintain the integrity of the research record. Additionally, these suggestions could enhance the efficiency of the investigative process, so that false alarms could be less resource-intensive.

## Abbreviations

COPE: Committee on Publication Ethics
